# Exploring the dynamic effect of economic growth on carbon dioxide emissions in Africa: evidence from panel PMG estimator

**DOI:** 10.1007/s11356-023-30108-4

**Published:** 2023-10-17

**Authors:** Delphin Kamanda Espoir, Regret Sunge, Frank Bannor

**Affiliations:** 1 School of Economics, University of Johannesburg, Johannesburg, South Africa; 2 Department of Economics, Munhumutapa School of Commerce, Great Zimbabwe University, Masvingo, Zimbabwe

**Keywords:** Economic growth, Environmental pollution, Cross-sectional dependence, Heterogeneity, Granger causality, Africa, Q53, Q54, Q56

## Abstract

The relationship between economic growth and environmental pollution continues to attract significant research interest for researchers, practitioners, and policymakers all over the globe. Theoretically, the environmental benefit of economic growth should be greater than its negative externality with higher level of development. However, from the African perspective, countries with higher economic performances often face several environmental challenges, which raises the doubt whether economic growth helps or constrains environmental quality improvement. Under the environmental Kuznets curve (EKC) hypothesis, this study re-examined the effect of economic growth on CO2 emissions conditional on the dynamics of urbanization, renewable energy, and good governance across 47 African countries using panel data from 1996 to 2019. We employ panel cointegration tests to establish whether there is a long-run equilibrium relationship among our variables. We also apply pooled mean group ARDL (PMG-ARDL) techniques and the Dumitrescu-Hurlin causality test to determine the long- and short-run effects of economic growth, urbanization, renewable energy consumption, and good governance on CO2 emissions. The results from the PMG estimator validate the EKC hypothesis since a 1% surge in GDP per capita increases emissions by 0.61% in the long run, while a 1% increase in its square decreases emissions by 0.03%. In the short-run, economic growth does not exercise any significant effect on emissions. Furthermore, results indicate a significantly negative and positive long-run effect of renewable energy and governance, respectively. Finally, our causality test shows bidirectional relationship between CO2 emissions and all the explanatory variables. Henceforth, we provided policy implications based on the study’s results.

## Introduction

As climate change and global warming continue to inflict socio-economic challenges globally, the nexus between greenhouse gas (GHG) emissions and economic growth has moved to the front of the environmental sustainability and development debate. Current deliberations on environmental degradation, climate change, and sustainable development are spearheaded by the 2016 Paris Agreement. This is a product of the Framework Convention on Climate Change (UNFCCC) and the Intergovernmental Panel on Climate Change (IPCC). These policy establishments are geared to guide policy on climate change and development. They also complement the achievement of Sustainable Development Goal (SDG) 13 which treats combating climate change and its effects as an urgent matter (United-Nations 2015). Within this domain, dialogue on economic growth and greenhouse emissions, usually proxied by CO2 emissions, is topical.

The increased attention reflects the existence of a complicated interdependence and possible trade-off between environmental degradation and economic growth. On one hand, it has been shown that economic growth is positively driven by energy consumption (Chang et al. 2015; Iftikhar ul Husnain et al. 2021; Khan et al. 2023; Mutascu 2016; Wang et al. 2023). On the other, energy consumption is known as the major source of greenhouse emission (Afriyie et al. 2023) which in turn reduces economic growth (Aye and Edoja 2017). The effect of emissions in Africa is projected to be relatively severe for two reasons. First, most countries are still developing. As such, the demand and consumption for fossil energy and CO2 emissions are expected to continue an upward trend (Hamilton and Kelly 2017). Second, Africa does not have the required finance to greener technologies and lack effective regulations to reduce energy-related CO2 emissions (Tajudeen 2015).

In view of this, we draw attention to the nexus and causality between economic growth and CO2 in Africa, conditional on key variables mediating this relationship (urbanization, renewable energy, industrialization, etc.). We appreciate existing related studies on this relationship focusing on Africa, whose evidence is mixed. We note two cluster of studies. The first simply examines the impact of economic growth on CO2 emissions by testing the environmental Kuznets (EKC) hypothesis. The EKC hypothesis is supported by some (including Adzawla et al. 2019; Arouri et al. 2012; Kasperowicz 2015; Saba 2023) and rejected by others (including Abid 2016; Aye and Edoja 2017; Demissew Beyene and Kotosz 2020). One major drawback we pick from these studies is that they impose a unidirectional causality from economic growth to CO2 emissions and ignore the reverse causality. The second cluster (including Chekouri et al. 2021; Dogan et al. 2020; Odhiambo 2017; Omri et al. 2015; Zaidi and Ferhi 2019) examines the causality relationship between the two. Findings are diverse mainly according to countries, regions, and econometric estimation techniques. Furthermore, in previous decades, less attention has been given to CO2 emissions generated on the African continent because of the region’s low socioeconomic development and weak governance institutions. However, with the recent rapid growth of the total and urban population, and the need for a transformation from an agrarian society into an industrialized society to spearhead economic growth, the African continent is expected to consume more energy. This is because the total and urban population continue to increase the demand for gas, oil, and coal, among others is increasing, which will consequently contribute to CO2 emissions. With this describing phenomenon, it has become crucial to investigate the dynamic effect of economic growth on CO2 emissions and also study the contributing effect of urbanization, renewable energy consumption, and governance across African countries.

Most existing studies investigated the issues related to economic development and the environment using various panel methods within the EKC hypothesis framework. But those studies emphasize less the role of urbanization, renewable energy, and most importantly institutional governance. Hence, we seek to make an important contribution to literature. We incorporate two key variables that should be addressed in the economic growth-CO2 relationship in the African context-—institutional governance and urbanization. Controlling for governance when studying the impact of economic growth on CO2 emissions is significant since governments are at the center of public affairs; they can pass laws to protect public health and create regulations to enforce them. The essence of governance is to protect its citizens and to preserve the environment and attendant resources from ecological footprints and hazardous wastes. The quantitative effects of these government environmental ordinances remain a prior unclear and need to be studied (Adekunle 2021).

For instance, Adedoyin et al. (2020) used the pooled mean group-autoregressive distributed lag (PMG-ARDL) model to investigate the relationship between environmental sustainability, urbanization, and agro-economic performance in 12 SSA countries; Musibau et al. (2020) employed the quantile–quantile regression to investigate the role of green energy and energy innovation in the environmental Kuznets curve; Wang and Dong (2019) used the augmented mean group (AMG) estimator to investigate the impact of economic growth and non-renewable and renewable energy consumption. While an analysis by Afriyie et al. (2023) controlled for urbanization based on a sample data of 37 African countries, they failed to consider governance among the selected control variables.

Urbanization, according to the African Development Bank (AfDB) (2022), is one the most profound transformations that the African continent will undergo in the twenty-first century. We find some studies on urbanization and CO2 emissions in Africa (Erdoğan et al. 2022; Hussain et al. 2022) without controlling for institutional governance. The importance of combining the two can be deduced from (Enserink and Koppenjan 2007) who find the potential environmental gains through good governance in sustainable urbanization strategy policy in China. The study acknowledged that urbanization brings environmental complexities which affect governance. It is therefore important to investigate the environmental effects of urbanization and governance in Africa given the increased rate of urbanization and a highly sensitive and fragile governance system.

The rest of the study is structured as follows: the “CO2 emissions in Africa: a brief overview” section gives a brief overview of CO2 emissions in Africa; the “Literature review” section presents the study’s theoretical framework as well as the empirical literature on the CO2-growth nexus. The methodology and estimation techniques are detailed in the “Data and methodology” section, results are presented and discussed in the “Results and discussion” section, while the “Conclusion and policy implications” section concludes and give recommendations.

## CO2 emissions in Africa: a brief overview

Greenhouse gas (GHG) emission is a major concern all around the world, yet the consequences of its effect on quality of life and climate vary by region (Gavurova et al. 2021). Carbon dioxide (CO2) emissions are often the most significant source of GHG emissions, accounting for around 70% of total GHG emissions (Espoir and Sunge 2021). While developed nations have dominated historical global CO2 emissions, emerging economies’ rapid increase in energy demand may push their absolute emissions above those of industrialized countries. In 2005, it was estimated that industrialized countries contributed for roughly 40% of global CO2 emissions, developing countries for about 56%, and aviation and maritime transport accounted for the remaining 4% (Kijewska and Bluszc 2016). Africa is one of the continents where nearly half of the population does not have access to electricity, and more than 60% of the population still cooks with traditional biomass (International Energy Agency 2019).

Despite the fact that energy access is a key priority, extending modern energy services in most African countries under a business-as-usual policy scenario would result in significant increases in both primary energy supply and CO2 emissions by 2030 (Hamilton and Kelly 2017). This is due to the fact that present regulations in many African nations will not be able to avoid such increases in energy-related CO2 emissions (Tajudeen 2015). More so, although many electricity-related initiatives focus on large-scale hydropower, natural gas and increasing consumption of oil have become significant contributors of CO2 emissions. Countries such as Botswana, Kenya, and Senegal have already witnessed growing emissions, owing mostly to increased coal usage, leading to a 0.7–1% yearly increase in CO2 emissions. More so, in both absolute and per capita terms, Africa’s CO2 emissions from fossil fuels are modest. However, Africa’s total carbon emissions have risen 12-fold since 1950, reaching 311 million metric tons in 2008. Emissions from all fuel sources have increased in the African area over time, with liquid and solid fuels accounting for about 35% of total emissions and gas fuels accounting for 16.9% (Andres et al. 2011).

A limited number of countries are mainly responsible for African emissions from fossil fuels and cement manufacturing. South Africa accounts for 38% of the continental total, while Egypt, Algeria, Nigeria, Libya, and Morocco together account for 46%. Altogether, these six countries have yearly CO2 emissions above 10 million metric tons. At the same time, four African countries, Libya (2.53).

South Africa (2.39), the Seychelles (2.22), and Equatorial Guinea (1.99), have CO2 emissions per capita that exceed the world average of 1.3 metric ton of carbon per year (Andres et al. 2011). In addition, emissions from waste disposal in cities in developing countries are expected to grow significantly in the near future, with methane released by dumpsites and landfills accounting for the majority of these emissions (Friedrich and Trois 2011). Emissions from garbage disposal are projected to represent 8.1% of total GHG emissions in Africa.

**Fig. 1:**
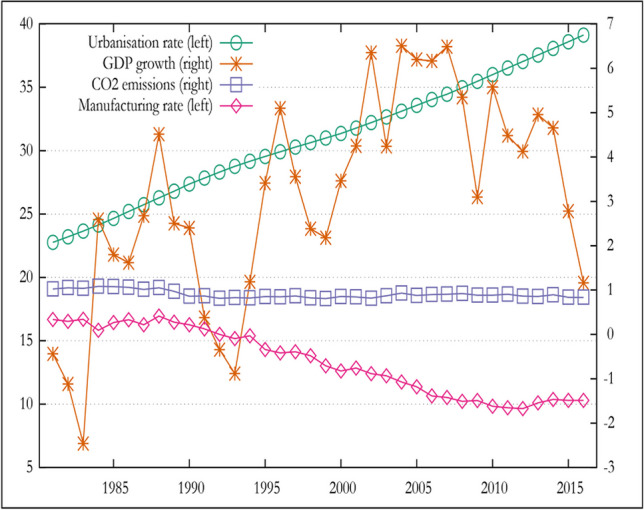
SSA trend of urbanization rate, GDP growth, manufacturing value added, and CO2 emissions, 1981–2016. Source: authors’ computation using World Bank data

**Fig. 2:**
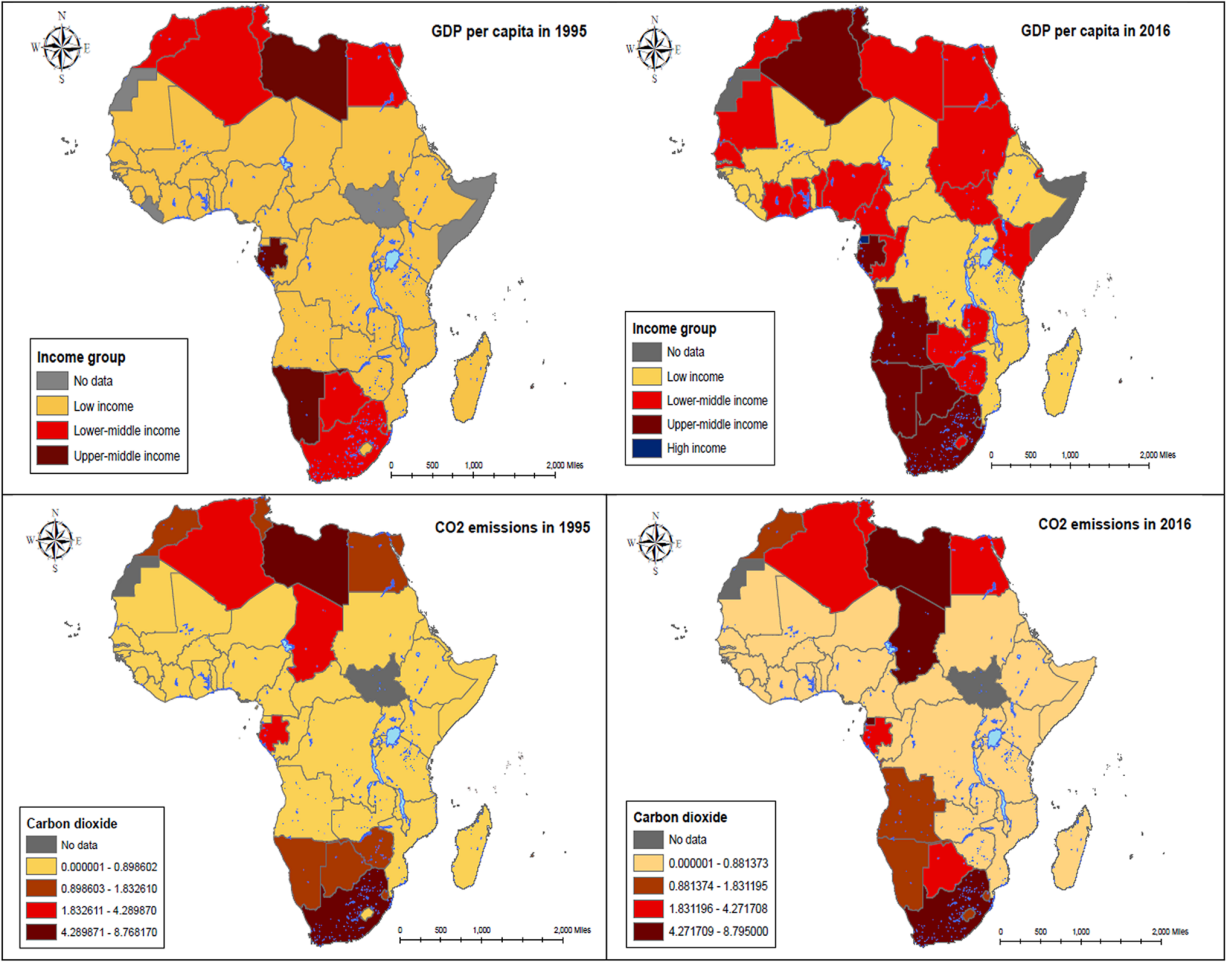
GDP and carbon emissions per capita across African countries in 1996 and 2016

While the population of cities is rapidly growing in Africa, with around 54% predicted to live in urban areas by 2030 (Antonel and Chowdhury 2014), the African continent’s fast population growth, industrialization, and urbanization are not without environmental effects (see Fig. 1). For example, it can be seen in Fig. 2 that there is a co-movement between CO2 emission and GDP growth from 1981 to 2016. At the same time, urbanization rate has increased over time. This confirm the Kaya identity–total carbon emissions are influenced by economic growth, the intensity of energy consumption, and population increase (Kaya and Yokoburi 1997).

## Literature review

### Theoretical framework

In this section we summarize the theoretical underpinnings connecting CO2 emissions to economic growth. A consensus in literature is that this connection is rooted to the environmental Kuznets curve (EKC) hypothesis. The EKC hypothesis was initially confirmed by Simon Kuznets (1955) when examining the nexus between economic growth and income inequality. Kuznets (1955) concluded that initial stages of economic growth are associated with increasing income inequality up to a maximum point, after which continuous economic growth reduces inequality (Saba 2023). Espoir and Sunge (2021) credits the application of the EKC hypothesis to environmental economics to the works of Meadows et al. (1972), Jahoda (1973), Grossman and Krueger (1995), and Shafik and Bandyopadhyay (1992). Their investigation were in line with that of Kuznets. In the end it was concluded that the effect of economic growth on CO2 emissions varies with scale over time. During early stages, growth is associated with increasing emissions. However, as economies of scale, income, and innovation increase, higher output becomes environmentally sustainable. Accordingly, an inverted U-shaped association between environmental degradation and economic growth was established. This finding has been widely accepted as the environmental Kuznets curve (EKC) hypothesis.

To explain this relationship, Grossman and Krueger (1995) specified environmental degradation as a function of income per capita, the square of income per capita as follows:

1 $${ln\left(\frac{E}{P}\right)}_{it}={\delta }_{i}+{\varphi }_{t}+{\beta }_{1}{lnln \left(\frac{GDP}{P}\right) }_{i,t}+{\beta }_{2} {(ln\left(\frac{GDP}{P}\right))}_{i, t }^{2}+{\varepsilon }_{i,t}$$

where $ln$ is natural logarithm, *E* is CO2 emissions, *P* is population, and ${\delta }_{i}$, ${\varphi }_{t}$ are elasticities which changes over cross sectional units $i$ and time $t$. The fixed effect assumes that for any given income level, all states face the same income elasticity. The time intercepts captures time-varying omitted variables and shocks common to all states and units. ${\beta }_{j}$= 1, 2 are the parameters to be estimated. The EKC hypothesis is said to hold if ${\beta }_{1}$ is positive while ${\beta }_{2}$ is negative. The income level where emissions are maximized is given as

2 $$\tau =exp\left(\frac{-{\beta }_{1}}{2{\beta }_{2}}\right)$$

Usually, empirical examinations based on the EKC hypothesis add control variables to complete the effect of growth on CO2 emissions such that

3 $${ln\left(\frac{E}{P}\right)}_{it}={\delta }_{i}+{\varphi }_{t}+{\beta }_{1}{lnln \left(\frac{GDP}{P}\right) }_{i,t}+{\beta }_{2} {(ln\left(\frac{GDP}{P}\right))}_{i, t }^{2}+{\sum }_{{\beta }_{=3}}^{J} {X}_{it}+{\varepsilon }_{i,t}$$

where $X$ is a vector of additional explanatory variables up to $J$.

In the same spirit, we include urbanization (Mentel et al. 2022; Adedoyin et al. 2020; Afriyie et al. 2023), renewable energy consumption (REN) as percentage of total final energy consumption (Bosah et al. 2021; Afriyie et al. 2023), and governance (Espoir and Sunge 2021).

Several arguments have been raised to support the existence of the EKC hypothesis. According to Lu (2017), in the early stages of economic growth, industrial production depends more on fossil fuel–generated energy which emit higher greenhouse emissions. This usually happens in low-income and pre-industrial economies. As growth advances, higher incomes permit investment in production technologies which reduces greenhouse emissions (Stern 2004; Abid 2016). This phase is associated with post-industrial high-income economies. However, over the years, the EKC hypothesis has developed into an empirical question. Other recent studies (Arouri et al. 2012; Kasperowicz 2015; Adzawla et al. 2019) confirm it while others (including Abid 2016; Aye and Edoja 2017; Demissew Beyene and Kotosz 2020) reject it. Unlike the causality from economic growth to CO2 emissions, there is no clear theory to explain the reverse causality. However, several reasons have been raised.

The economic growth effects of CO2 emissions are usually linked to the environmental consequences of pollution. Emissions have been the major cause of climate change and global warming. Globally, mean surface temperature increased by approximately 1 °C (1.8 °F) since 1900. As a result, the global economy has been experiencing environmental imbalance. This has increased the vulnerability of economic growth to persistent floods, cyclones, droughts, and diseases; decreases agricultural productivity and food production; and increasing diseases and their spread, among others (Aye and Edoja 2017). These hazards have cross-cutting adverse effects on economic growth. As the Economist Intelligence Unit (EIU) (2020) reports, climate change effects may lead to a loss of 3% of global gross domestic product (GDP) by 2050, with Africa set to lose more (4.7%).

### Empirical literature review

Several studies have examined the causality relationship between economic growth and CO2 emissions involving regions and countries in Africa. Odhiambo (2017) applied a dynamic-panel Granger-causality approach on data for the period 1986–2013 for 10 SSA countries. Results indicate that there is a unidirectional causal relationship running from economic growth to CO2 emissions in both the short run and long run. However, Zaidi and Ferhi (2019) offer different evidence of bi-directional causality evidence for SSA from a dynamic GMM simultaneous-equation estimator for almost the same period, 2000–2012. Dogan et al. (2020) draws our attention to a different dimension in the relationship in Africa. Instead of examining the CO2 causality on economic growth, they did so on total factor productivity. Also, their evidence is not based on linear causality but rather on a nonlinear nonparametric quantile causality approach. The study documents bidirectional causal ordering for nearly all the sample countries.

However, in a related study on Middle East and North African Countries (MENA), Omri et al. (2015) find evidence for bi-directional causality relationship between the two over the period 1990–2011. A study on BRICS countries^1^ by Cowan et al. (2012) suggests that the existence and direction of causality are country specific. The study found causality from GDP to CO2 emissions in South Africa, reverse causality from CO2 emissions to GDP in Brazil, bi-directional causality for Russia, and no causality for in India and China. Country evidence is also available for Algeria. Chekouri et al. (2021) employed the wavelet power spectrum (WPS) and Breitung and Candelon (2006) approaches for the period 1971–2018. WPS results suggests co-movement between growth and CO2 emissions, while causality tests confirm a one-way causality from growth to CO2 emissions in both the short and long run.

More recently, Khan et al (2023) examined how poverty, income inequality, and energy consumption affect carbon dioxide emission in the Belt and Road Initiative using data spanning 1996 to 2018. Results from the generalized method of moments (GMM) revealed poverty, inequality, and energy consumption increase CO2 emissions. However, electricity was found to have favorable environmental effects. Their results also confirm the EKC hypothesis. Another study (Kongkuah et al. 2022) with interest in the Belt and Road Initiative used a different methodology to record contrasting results. After using Fourier ADF (FADF) unit root test, the Fourier ADL, Gregory-Hansen cointegration tests, the time-varying bootstrap causality test, among other methods, no evidence for the EKC was found.

With more interest in the effects of military expenditure on CO2 emissions in South Africa, Saba (2023), after employing dynamic ARDL simulations, found that militarization had an insignificant positive short run, but a significant and negative impact on CO2. The results failed to validate the treadmill theory of destruction in South Africa. However, after finding that economic growth had a positive and significant short run effect and a significant negative long run effect, the EKC was validated. Focusing on the moderating role of institutional quality, Chinonye et al. (2022) employed ARDL and quartile regression approaches to dismiss the EKC hypothesis in Nigeria. Instead, they found an N-shaped relationship between CO2 emissions and economic growth in both the short run and the long run. Moreover, Adekunle (2021) investigated the role of governance and good institution on the search for environmental sustainability using the dynamic system generalized method of moment estimator and the data of 53 African countries from 1996 through 2017. Results suggested a positive relationship between the rule of law and regulatory quality and transformation to environmental sustainability.

Adedoyin et al. (2020) examined the relationship between urbanization, agro-economic performance, and environmental sustainability of 12 selected sub-Saharan African using the PMG ARDL. The study employed time-series cross-section data for the period spanning 1980–2014 and found that both urbanization and economic output have a significant impact on CO2 emissions. The study recommended the promotion of good agricultural practices to enhance the environmental sustainability in those selected countries. Contrary to previous research, this study employs the PMG estimator proposed by Pesaran et al. (1999) to consider a lower degree of heterogeneity. PMG ensures homogeneity as well as heterogeneity in the long-run coefficients and short-run coefficients, respectively, and it also allows error variances.

## Data and methodology

### Data

We employ panel data to investigate the dynamic effect of economic growth, urbanization, renewable energy, and governance with CO_2_ emissions. Our sample includes 47 African countries and data covers the period going from 1996 to 2019. CO_2_ emission per capita is used as the dependent variable and it is measured in metric tons. The independent variables in this study are as follows: the GDP per capita (current 2015 US $) is used as proxy for economic growth (Espoir and Sunge 2021); the size of the urban population taken as a share of the total population (URB). Similar studies such as Mentel et al. (2022), Adedoyin et al. (2020), and Afriyie et al. (2023) used the same proxy to investigate the impact of urbanization on CO_2_ emissions across various countries in the World; renewable energy consumption (REN) as percentage of total final energy consumption (Bosah et al. 2021; Afriyie et al. 2023). Time-series data of CO2 per capita, GDP per capita, urbanization, and renewable energy consumption are sourced from the World Development Indicators (WDI). Finally, the governance quality (GOV) is used to measure the impact of the quality of the African institutions on the environmental sustainability (Espoir and Sunge 2021).

We construct the governance index (GOV) using data from the World Governance Indicators (WGI) and the principal component analysis (PCA) technique. We employ the PCA to derive the principal index of six institutional quality indicators ((political stability and absence of violence (PV), government effectiveness (GE), control of corruption (CC), voice and accountability (VA), regulatory quality (RQ), and the rule of law (RL)). In constructing the composite index of governance indicators, we first start by collecting the residuals from the regression of a particular composite index of the institutional quality (see Espoir and Sunge 2021). Then, the residuals obtained from each regression are aggregated through PCA. According to the literature, the PCA is a procedure that takes high dimension sets of indicators and transforms them into novel indices that capture information on a different dimension and are mutually uncorrelated. Then, to obtain an aggregated GOV, the first eigenvectors (factor loadings) from the PCA could be employed as the required weights. Thus, the linear combination of the index was calculated as follows:

4 $${GOV}_{i,t}={\phi }_{1}{PV}_{i,t}+{\phi }_{2}{GE}_{i,t}+{\phi }_{3}{CC}_{i,t}+{\phi }_{4}{VA}_{i,t}+{\phi }_{5}{RQ}_{i,t}+{\phi }_{6}{RL}_{i,t}$$

where ${\phi }_{1}$, ${\phi }_{2}$, ${\phi }_{3}$, ${\phi }_{4}$, ${\phi }_{5}$, and ${\phi }_{6}$ are the eigenvectors (factor loadings) obtained from the PCA and *PV*, *GE*, *CC*, *VA*, *RQ*, and *RL* are subscriptions of the six indicators of governance. Table 1 presents the results of the governance index (GOV) variable. For this variable, we only consider the component that obtained an eigenvalue greater than 1 and those eigenvectors associated with variables whose factor loading exceeded 0.30 in absolute value. The analysis of these results revealed that one single factor (eigenvalue = 4.845) from the PCA entirely explains 80.7% of the total variance. Consequently, we retain only the first component for GOVI, as it retained approximately 80% of the variance of the initial data.

**Table 1:** Principal component analysis results

PCA results (panel A)	Eigenvalue	Difference	Proportion	Cumulative
Components				
Component 1/(dimension 1)	4.845	4.433	0.807	0.807
Component 2/(dimension 2)	0.411	0.077	0.068	0.876
Component 3/(dimension 3)	0.334	0.089	0.055	0.931
Panel (B): PCA eigenvectors results Variable	Component 1	Component 2	Component 3	Unexplained
PV	0.371	0.871	-0.132	0.013
GE	0.424	− 0.323	-0.273	0.060
CC	0.411	− 0.111	-0.326	0.140
VA	0.386	− 0.002	0.887	0.012
RQ	0.412	− 0.350	0.014	0.126
RL	0.440	0.010	-0.113	0.055

Source: authors’ illustration from PCA results

### Model specification

In examining the dynamic effect of economic growth, urbanization, renewable energy, and governance with CO_2_ emissions, we deploy the panel PMG-ARDL estimation technique proposed by Pesaran et al. (1999). This technique has been recommended for panel data exhibiting homogeneity as well as heterogeneity in the long-run and short-run regression coefficients, respectively, but also variances in predicted error. Furthermore, PMG estimator corrects the problem of endogeneity caused by endogenous regressors by selecting the most appropriate lag structure for both the dependent and independent variables. Finally, PMG estimator offers three possibilities to estimate non-stationary dynamic panel data when the estimated coefficients are heterogeneous between groups (the pooled mean group (PMG) estimators, the mean group (MG), and the dynamic fixed effects (DFE)). In this study, we considered the case of PMG estimator where the long-run effects are constrained to be equal across all units and the short-run coefficients are accepted to vary across panels, and the DFE estimator where all coefficients are constrained to be equal across units. We finally preferred the PMG because our panel data fail to reject the homogeneity restriction with a statistically insignificant probability value of 1.000 obtained from the Hausman test. For the model specification, in this current study, we followed the specification of Afriyie et al. (2023) to accomplish the objectives. Unlike these two studies and for most research on the effects of economic growth, urbanization, and renewable energy on CO_2_ emissions in Africa, we also added an index of governance in our model as control variable. Thus, the model is expressed as

5 $${\mathrm{CO}}_{2} = f(\mathrm{GDP};\mathrm{ URB};\mathrm{ REN};\mathrm{ GOV})$$

In Eq. (1), CO_2_ = the stock of carbon dioxide generated from the burning of fossil fuels and the manufacture of cement; GDP = the gross domestic product per capita (current 2015 US $); URB = the urban population size as a share of the total population; REN = the renewable energy consumption (% of total final energy consumption); and GOV = the governance index generated using PCA. In our model, the selected variables are adjusted by considering their logarithmic form. In so doing, the logarithmic form of the variables allows us to interpret the estimated coefficients as percentage change or elasticity. An additional merit of considering the variables into their logarithmic form is to minimize the outliers of time series. This procedure prevents to get explosive estimated marginal effects. Given this, we replace the vector $$X$$ in Eq. (3) with URB, REN, and GOV such that our econometric models becomes

6 $$ln{CO2}_{i,t}= {\beta }_{0}+{\beta }_{1}\left(\mathrm{ln}{GDP}_{i.t}\right)+{\beta }_{2}\left(\mathrm{ln}{SQGDP}_{i,t}\right)+{\beta }_{3}\left(\mathrm{ln}{URB}_{i,t}\right)+{\beta }_{4}\left(\mathrm{ln}{REN}_{i,t}\right)+{\beta }_{5}\left(\mathrm{ln}{GOV}_{i,t}\right)+{\varepsilon }_{i,t}$$

With the introduction of the squared GDP variable in Eq. (2), the EKC hypothesis is confirmed with positive *β*1 and negative *β*2 (Espoir and Sunge 2021). Applying the PMG ARDL estimator framework (Pesaran et al. 1999) to the variables of this study as specified in Eq. (2), we get our estimated model representation as follows:

7 $$\begin{array}{c}\Delta ln{CO2}_{i,t}= A+\delta \left(ln{CO2}_{i,t-1}\right)+ {\varphi }_{i}\left( {\sum }_{i=1}^{p}\Delta ln{CO2}_{i,t-i}\right)+{\vartheta }_{i}({\sum }_{i=1}^{p}\Delta ln{GDP}_{i,t-i})\\ +{\sigma }_{i}\left({\sum }_{i=1}^{p}\Delta ln{SQGDP}_{i,t-i}\right)+{\alpha }_{i}\left({\sum }_{i=1}^{p}\Delta ln{URB}_{i,t-i}\Delta ln{URB}_{i,t-i}\right)+{\gamma }_{i}\left({\sum }_{i=1}^{p}\Delta ln{REN}_{i,t-i}\right)\\ \begin{array}{c}+{\pi }_{i}\left({\sum }_{i=1}^{p}\Delta ln{GOV}_{i,t-i}\right)+{\beta }_{0}\left(\mathrm{ln}{CO2}_{i.t-1}\right)+ {\beta }_{1}\left(\mathrm{ln}{GDP}_{i.t-1}\right)+ {\beta }_{2}\left(\mathrm{ln}{SQGDP}_{i.t-1}\right)\\ +{\beta }_{3}\left(\mathrm{ln}{URB}_{i.t-1}\right)+ {\beta }_{4}\left({REN}_{i.t-1}\right) {\beta }_{5}\left({GOV}_{i.t-1}\right)+ {\varepsilon }_{i,t}\end{array}\end{array}$$

In Eq. (4), $\delta$= the coefficient of the past lagged value of the dependent variable; ${\varphi }_{i}$, ${\vartheta }_{i}$, ${\sigma }_{i}$, ${\alpha }_{i}$, ${\gamma }_{i}$, and ${\pi }_{i}$ are the short-run estimated coefficients, while parameters from ${\beta }_{0}$ to ${\beta }_{5}$ are the long-run coefficients. The PMG estimator is valid if the error correction term (ECT) is negative and larger than − 2.

## Results and discussion

### Descriptive statistics and correlation

By employing the PMG estimator for this study, we started by analyzing the statistical properties of the variables. Firstly, Table 8 in the Appendix provides the list of countries included in our sample. Secondly, the summary of the descriptive statistic is presented in Table 2. The total number of observations for the entire panel was found equal to 1124, which correspond to data of 47 countries with a timeframe of 24 years (1996–2019) for each country. In terms of CO2 emissions, the average stock is 1.2667 metric tons per capita, with a minimum stock of 0.0162 and a maximum stock of 11.204. We found an average GDP per capita equal to $2027.247 for the full-panel with the minimum of $102.598 and maximum of $22942.58. For urbanization, the study recorded an average value of 39.76% of the population, with the minimum of 7.21% and maximum of 88.56%. Moreover, it can also be observed in our findings that the average share of renewable energy consumption (% of total final energy consumption) is 61.03% with the minimum value 0.0589% and the maximum value of 98.34%. Lastly, for governance index, the average, minimum, and maximum, values of 0.1862, − 4.6853, and 5.3472 were documented, respectively. It is essential to indicate that the study samples are positively skewed, suggesting that the series is skewed to the right.

**Table 2:** Descriptive statistics

	CO_2_	GDP	URB	REN	GOV
Mean	1.2667	2027.247	39.7648	61.0343	0.1862
Median	0.3225	796.922	38.7805	73.2751	0.1855
Minimum	0.0162	102.598	7.2110	0.0589	− 4.6853
Maximum	11.204	22,942.58	88.559	98.3426	5.3472
Std. dev	2.1922	2944.815	17.1879	30.6444	2.094
Kurtosis	9.2734	15.3272	2.6513	2.0954	2.7004
Skewness	2.6211	3.0927	0.3980	− 0.6906	0.3708
Shapiro-Francia *W* test	0.5844	0.6258	0.9780	0.8785	0.9793
Probability	0.000***	0.000***	0.000***	0.000***	0.000***
Observations	1128	1128	1128	1128	1128

^***^, **, and * represent 1%, 5%, and 10% level of significance

Figure 2 presents the average GDP and CO2 emissions per capita for each country in Africa for the year 1996 and 2019. For both periods, we observe dramatic grouping among African countries, especially between the Western and Central African regions against the Northern and Southern regions. In general, the significant differences in the level of economic development and carbon emissions distribution across African countries prompt us to consider the heterogeneous effect when testing for feedback impact between carbon emission and output growth.

**Fig. 3:**
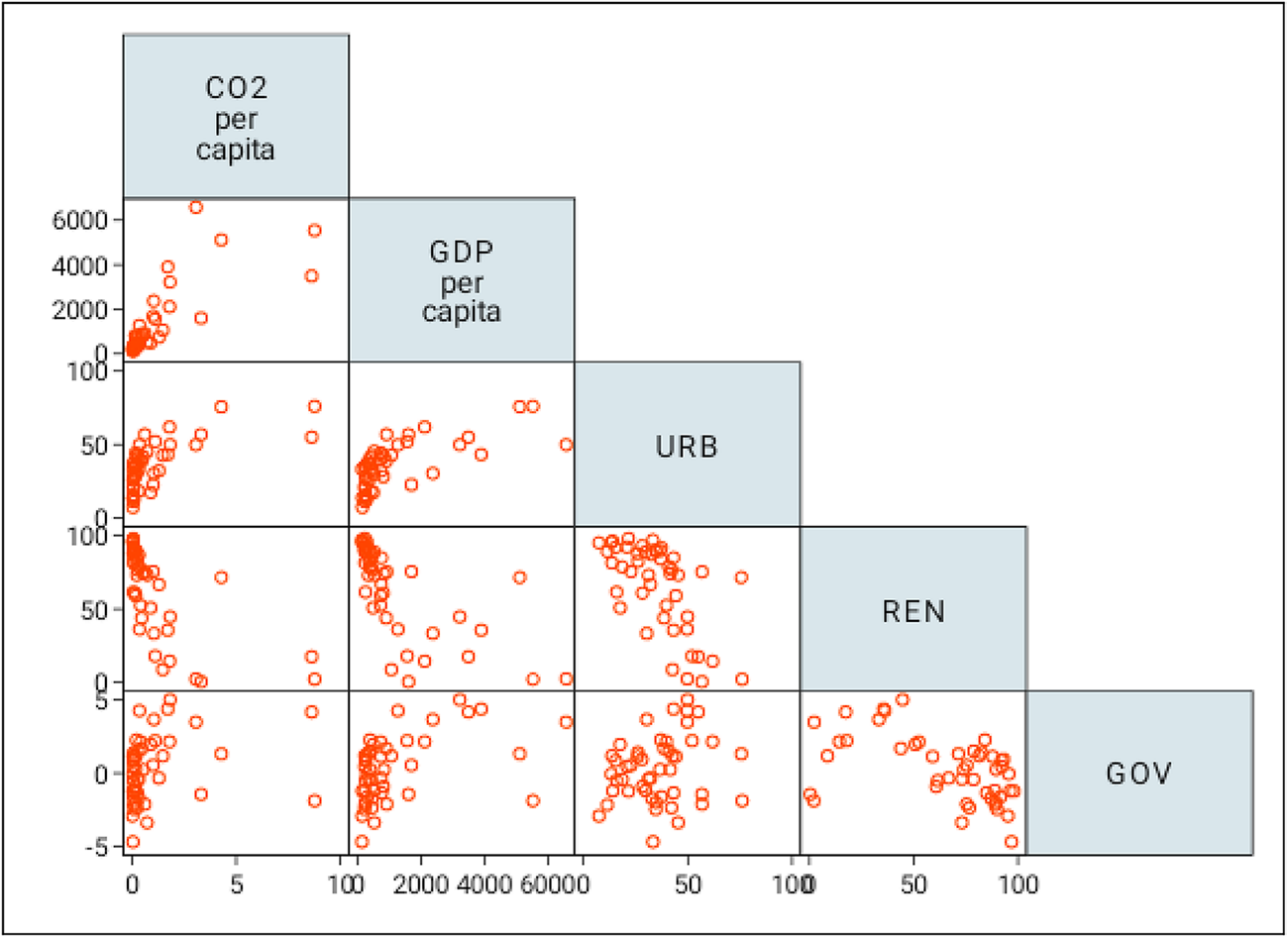
Correlation matrix between CO2 emissions and the independent variables

We have also conducted the correlation matrix analysis to examine the link between our sampled variables. Figure 2 presents the results of the half correlation matrix where the “dependent” variable is moved to the end of the variable list. The findings show a positive and statistically significant link between economic growth, urbanization, governance, and carbon emissions (first column of Fig. 3). Additionally, the findings reveal a negative and statistically significant relationship between renewable energy consumption and carbon emissions. The positive relationship between urban population and environmental quality is now well established in several African countries. Urban centers in several African countries have seen gradual improvements in their education and health systems as well as better infrastructural and industrial development, which have, as a result, served as pull factors for rural dwellers to migrate to urban centers (Afriyie et al. 2023). Consequently, our findings suggest that a rise economic growth, urban population, and governance quality contributes enormously to increasing carbon dioxide emissions which consequently decreases the quality of the environment in sub-Saharan Africa. Besides, our findings imply that an increase in green energy consumption improves the environmental quality across countries in Africa due to the negative relationship between the two variables. Nevertheless, the correlation matrix analysis is not enough to draw conclusions for the dynamic effects of our independent variables on CO2 emissions. Hence, an additional step is taken by conducting a more robust regression analysis using the PMG-ARDL technique.

### Cross‑sectional dependence and panel unit root tests

Before we perform the stationarity test, we start by investigating the presence or not of cross-section dependence using the Pesaran (2004) cross-dependence (CD) test. The null hypothesis of the test indicates no cross-sectional dependence. If the *p*-value of the Pesaran CD statistics is less or equal to the standard statistical levels, the null hypothesis is rejected. Table 3 presents the results of the CD test for both the full panel and the variables take individually. The results indicate that the null hypothesis of no cross-sectional dependence in the panel is strongly rejected at the 1% level of significance since the *p*-value of 0.0000 is less than the statistical significance level of 0.05. Similarly, the results of the variables taken individually indicate that the null hypothesis of no cross-sectional dependence is rejected for all the variables except governance (GOV).

**Table 3:** Cross-sectional dependence test results

Residual cross-section dependence test (full panel)						
Pesaran (2004) CD test	Statistic	*p*-value				
	81.09	0.0000				
Cross-section dependence test (individual variable)						
	Variable	ln CO_2_	lnGDP	URB	REN	GOV
	Statistic	41.14***	130.05***	115.72**	45.63***	-1.18

^***^ and ** indicate 1 and 5% level of significance

Given that the results of the Pesaran (2004) CD test indicate the presence of cross-sectional dependence in the data, all panel unit root tests from the first generation are inconsistent as these tests are developed under strict assumption of cross-sectional independence. Thus, we then proceeded by applying specific panel unit root tests due to the positive results of cross-sectional dependence in the data. We employed two second-generation panel unit root tests. The cross-sectionally augmented IPS or CIPS panel unit root test was suggested by Pesaran (2007). By allowing for cross-section dependence in testing for panel unit root, the CIPS test augments the conventional augmented Dickey-Fuller (ADF) regression with the cross-unit averages of lagged levels and first differences of the individual series. In so doing, the estimates are obtained for both the cross-country augmented ADF (CADF) statistics and the CIPS. Mathematically, the CADF regression equation is presented as follows:

8 $$\Delta {Q}_{i,t}={Z}_{i,t}{\varnothing }_{i}+{\rho }_{i}{Q}_{i,t-1}+{\sum }_{s=1}^{{j}_{i}}{\theta }_{i,s}\Delta {Q}_{i,t-s}+{\pi }_{i}{\underline{Q}}_{i,t-1}+{\sum }_{s=0}^{{j}_{i}}{\varphi }_{i,s}\Delta {\underline{Q}}_{i,t-s}+{u}_{i,t}$$

where ${\underline{Q}}_{t}$ represents the cross-country average of ${Q}_{i,t}$; $\left({\underline{Q}}_{t}=\frac{1}{N}{\sum }_{i=1}^{N}{Q}_{i,t}\right).$ The null hypothesis to be tested is that each variable contains a unit root (H0:${\rho }_{i}$ = 0) for all country $i$, against the alternative hypothesis, indicating that at least one of the variables in the panel is stationary (H1: ${\rho }_{i}$ < 0), for at least one of the country $i$. In deciding whether the null hypothesis should be rejected or not, the CIPS statistic is used and calculated as the average of the individual CADF statistics as follows:

9 $$CIPS=\frac{1}{N}{\sum }_{i=1}^{N}{TR}_{i}$$

where ${TR}_{i}$ denotes the *t* − ratio obtained from the OLS regression on the coefficient of ${\rho }_{i}$ in the above CADF regression in Eq. (8). Table 4 presents the CIPS and CADF panel unit root results. Results from both tests indicate that all the variables are integrated of order 1 and can be noted as I(1). The logical implication of this conclusion is that there could exist at least one long-run equilibrium relationship among the variables for the full sample, henceforth, a need to test for panel cointegration.

**Table 4:** Second-generation panel unit root test results

	CIPS results		CADF results	
Variables	At level I(0)	At first diff. I(1)	At level I(0)	At first diff. I(1)
lnCO_2_	− 1.985	− 4.371***	− 1.735	− 2.297***
lnGDP	− 2.899***	− 4.205***	− 2.542***	− 2.835***
lnSQGDP	− 2.797***	− 4.100***	− 2.472***	− 2.772***
lnURB	− -1.491	− 2.462***	− 1.408	− 2.166*
lnREN	− 2.089*	− 4.080***	− 1.917	− 2.443***
lnGOV	− 2.360***	− 2.118**	− 3.032***	− 2.964***

^***^, **, and * indicate 1, 5, and 10% levels of significance

### Panel cointegration results

We deployed two categories of panel cointegration test in studying the long-run equilibrium relationship between economic growth, urbanization, renewable energy, and governance with CO_2_ emissions. Specifically, we used Pedroni (1999) and the Westerlund (2007) cointegration test. First, the Pedroni (1999) test is based on the traditional Engle-Granger (EG) cointegration test. The test allows for heterogeneity between each panel unit. It also includes heterogeneity in the long run cointegrating vectors as well as in the dynamics. Hence, there is no reason to consider homogenous parameters across panel units. Pedroni’s test has 11 different statistics grouped into two different categories (within and between dimensions). If the majority of those statistics reject the null hypothesis of no cointegration, it then implies the variables under consideration have a long-run co-movement. Table 5 presents the panel cointegration results. As shown in this Table, 6 out of the 11 statistics significantly reject the null hypothesis of no cointegration. This result therefore suggested the existence of the co-movement between economic growth, urbanization, renewable energy, governance, and CO_2_ emissions in the long-run equilibrium. Indeed, this finding is in line with the results of Afriyie et al. (2023). These authors investigated the dynamic nexus between urbanization and industrialization with carbon emissions in 37 sub-Saharan Africa and found cointegration among these variables.

**Table 5:** Panel cointegration results

**Pedroni test**	Statistic	*p*-value	Weighted statistic	*p*-value
Panel v-statistic	− 2.851	0.997	5.091	1.000
Panel rho-statistic	0.945	0.827	− 13.679***	0.000
Panel PP-statistic	− 12.260***	0.000	− 4.175***	0.000
Panel ADF-statistic	− 1.591**	0.050	10.747***	
Group rho-statistic	− 5.701	1.000		
Group PP-statistic	3.118	0.999		
Group ADF-statistic	− 7.522***	0.000		
**Westerlund test**				
	Statistic	*p*-value		
${G}_{t}$	− 2.675***	0.049		
${G}_{a}$	− 0.870	1.000		
${P}_{t}$	− 18.536***	0.000		
${P}_{a}$	− 3.713***	0.000		

^***^, **, and * represent 1%, 5%, and 10% levels of significance

Second, the Westerlund (2007) test is an error-correction-based panel cointegration test. Unlike the Pedroni test, the Westerlund (2007) panel cointegration test is more robust as it copes with the issue of structural breaks and cross-sectional dependence endogenously. The Westerlund test assesses whether there exists an error correction for individual panel units or for the entire panel. The test is constituted of two categories of statistics with each category having two statistics. The two statistics of the first category are known as panel statistics (${P}_{t}$, ${P}_{a}$). They are both obtained by pooling information concerning the error correction term along the cross-sectional dimension of the panel. The two statistics of the second category are also known as the group mean statistics (${G}_{t}$, ${G}_{t}$). The decision to reject the null is taken based on the significance of the majority of the four statistics (see Mitić et al. 2017). The results of the test are contained also in Table 5. As can be seen from this table, three test statistics (${G}_{t}$, ${G}_{a}$, and ${P}_{a}$) out of four reject the null hypothesis of no cointegration. Both tests recommended that there is at least one long-run equilibrium relationship among CO2 emissions and the independent variables. Next, we turned our focus by estimating the short- and long-run dynamics before investigating the variable causality.

### Short- and long-run relationship results

Table 6 presents the results of the short- and long-run relationship between economic growth, urbanization, renewable energy, governance, and CO2 emissions for both PMG and DFE techniques. Before we present the results of our regressions, it is important to mention that we performed the MG estimator and compared the results to those of PMG technique using the Hausman test. According to Pesaran et al. (1999), if the slope homogeneity assumption persists notwithstanding its ability to provide consistent estimates of the long run coefficients’ average, the MG estimator will be ineffective and inefficient. Thus, the PMG estimator should be considered more suitable and appropriate if the slope coefficients seem to be homogeneous across all unis. The Hausman result indicates that the homogeneity restriction hypothesis could not be rejected with a probability value of 0.860, which is statistically insignificant. This finding suggests therefore that under the null hypothesis, the PMG estimator is more suitable and preferred over the MG^2^ estimator for this study.

**Table 6:** Results of the short- and long-run effect

	Panel PMG results		Panel DFE results	
Dep. variable: lnCO2	Coefficient	*p*-value	Coefficient	*p*-value
**Long-run estimates**				
lnGDP	0.6138***	0.0000	0.9362***	0.0000
lnSQGDP	− 0.0327***	0.0000	− 0.0538***	0.0020
lnURB	0.0022	0.4600	0.0088	0.2520
lnREN	− 0.0186***	0.0000	− 0.0223***	0.0000
lnGOV	0.0381**	0.0310	0.0186	0.5950
**Short-run estimates**				
ECT(− 1)	− 0.4076***	0.0000	− 0.2714***	0.0000
∆ lnGDP	0.3294	0.6230	− 0.3513***	0.0120
∆ lnSQGDP	− 0.0163	0.7410	0.0283***	0.0040
∆ lnURB	− 0.1739	0.3300	0.0416*	0.0930
∆ lnREN	− 0.0360*	0.0610	− 0.0161***	0.0000
∆ lnGOV	− 2.7820	0.1670	− 0.1267	0.2730
Observations	987	0.0400	986	
Hausman ${\chi }^{2}$ statistic	0.04	1.0000		

^***^, **, and * represent 1%, 5%, and 10% levels of significance

As highlighted above, we presented in Table 6 the results of the PMG and DFE estimator. Once more, a Hausman test was performed to determine which one between the two estimators should be considered for our study. With a probability of 1.000, the Hausman statistic fails to reject the null hypothesis, indicating that the PMG coefficients are the most appropriate. Hence, we focused our interpretation on the PMG coefficients for policy formulation. Looking at the results in Table 6, it is seen that the error correction term has recorded a coefficient of − 0.4076 and is statistically significant at the 1% level of significance. This implies a faster return to equilibrium in case of disequilibrium. The error correction term indicates convergence to equilibrium in the long run (cointegration) in case of a shock in the short run. In addition, a higher coefficient equates to a faster adjustment process. Furthermore, the findings indicate that GDP per capita has a positive and significant impact on CO_2_ emissions, while the square of GDP per capita has a negative and statistically significant effect on CO2 emissions in our sampled countries. In particular, the results reveal that a 1% increase in GDP per capita increases pollution by 0.61% in the long run. As the economies of Africa grow and develop (SQGDP), a 1% increase in GDP per capita reduces pollution by 0.03%. This suggests that the EKC hypothesis holds for the sample of African countries. This finding is in line with the work of Espoir and Sunge (2021), who used dynamic panel data models to investigate the impact of economic development on CO2 emissions in Africa and found evidence of the EKC hypothesis. In the short run, the results indicate that both GDP and SQGDP per capita do not exercise any harmful effect on the environment in Africa. The findings indicate that urbanization has a positive and negative insignificant impacts on CO_2_ emissions in our sampled countries for the long and short terms. This finding is similar to the study conducted by Afriyie et al. (2023) and Bosah et al. (2021). Our finding on the insignificant effect of urbanization can possibly be explained by the fact that more than 80% of urban residents in Africa who have access to electricity experience increasingly regular interruptions in power supply. This phenomenon could influence the degree at which urban residential energy use impacts carbon emissions. In addition, it is important to note that the relationship between urbanization and carbon emissions is complex and context specific. While urban areas may have higher levels of energy consumption, they also tend to be more densely populated, which can promote more efficient energy use. Urban areas may have greater access to renewable energy sources and may be more amenable to implementing energy efficiency measures. In contrast, our finding is different to that of Mahmood et al. (2020) and Wang and Dong (2019) who reported a positive and significant impact of urbanization on CO_2_ emissions in Africa.

Looking at the results of renewable energy consumption, we find that this variable has a negative and statistically significant effect on CO2 emissions. Specifically, a 1% increase in REN reduces pollution by 0.02 and 0.04% in the long and short terms, respectively, across African countries. These findings are in line with Afriyie et al. (2023), Dogan and Seker (2016), Espoir and Sunge (2021), Tong et al. (2020), and Zhou et al. (2018), who concluded that energy consumption reduces environmental quality. Given the global climate agenda, the Paris agreement on climate promotes renewable energy sources since they reduce CO_2_ and ensure energy security supply. With a huge energy deficit, low income, and crumbling infrastructure make most African countries fertile ground for green energy development. Henceforth, multilateral organizations and private investors should increase their support and investments in renewable energy development projects to decrease energy production costs. Additionally, African governments should also adopt a carbon tax policy that increases fossil fuel costs. Such a measure may be a practical step toward the development of renewable energy on the continent. In terms of governance, the results in Table 6 documented a positive and significant effect on CO2 emissions in the long run but impact carbon emissions negatively in the short run, even though the impact is not statistically significant. A plausible reason for this relationship could be attributed to the fact that many African countries have not established robust institutional frameworks for environmental governance. As a result, their policies may prioritize economic growth over environmental protection. The results of our study contrast with those of Espoir and Sunge (2021), who observed a significant and negative relationship between governance and CO2 emissions in Africa.

### Panel causality test results

We analyzed the direction of the short-run dynamics between economic growth, urbanization, renewable energy, governance quality, and CO2 emissions using the Dumitrescu-Hurlin causality test (Dumitrescu and Hurlin 2012). The Dumitrescu-Hurlin panel causality test is based on a bivariate model. It is important to mention that the aim of testing for the causality between two variables is to help policymakers in formulating useful policies toward improving the environmental quality in Africa. The results of the causality analysis are reported in Table 7. Firstly, we notice bidirectional or feedback relationship between economic growth and CO2 emissions. This implies that economic growth Granger causes the level of carbon emissions and vice versa. This finding is in line with several recent studies who reported bidirectional relationship between the two variables (Afriyie et al. 2023). Policy wise, the feedback results of the short-run effect between economic growth and CO2 emissions in Africa imply that environmental sustainability decisions should be taken considering productivity concerns and vice versa. Also, there should be a benefit from consumers/firms in developed states purposefully and meaningfully investing in developing countries with a corporate social responsibility (CSR) or corporate environmental responsibility (CER) framework. We further established a bidirectional relation between urbanization and CO2 emissions where a decrease (increase) in one is capable of causing a decrease (increase) in the other. Afriyie et al. (2023) found the same results for a sample size of 37 African countries. The causality between renewable energy and CO2 emissions is also bidirectional. Besides, our finding indicates a bidirectional causality between governance and CO2 emissions. Finally, renewable energy consumption and GDP per capita are also found to exercise a feedback relationship. This means that both variables may act as a complement to each other. This result suggests energy conservation policies implemented could potentially have a destructive impact on economic growth in across African countries. Similar finding is found to be reported by Afriyie et al. (2023).

**Table 7:** Pairwise Dumitrescu and Hurlin (2012) panel causality test

Null hypothesis	*W*-statistic	*Z*-bar statistic	*p*-value
lnGDP does not homogeneously cause lnCO_2_	3.3324	11.3066	0.000
lnCO_2_ does not homogeneously cause lnGDP	3.2274	10.7977	0.000
lnURB does not homogeneously cause lnCO_2_	4.0121	14.6016	0.000
lnCO_2_ does not homogeneously cause lnURB	7.7422	32.6839	0.000
lnREN does not homogeneously cause lnCO_2_	2.1883	5.7605	0.000
lnCO_2_ does not homogeneously cause lnREN	1.9065	4.3944	0.000
lnGOV does not homogeneously cause lnCO_2_	4.1915	15.4713	0.000
lnCO_2_ does not homogeneously cause lnGOV	6.6720	27.4960	0.000
lnGDP does not homogeneously cause lnURB	9.6057	41.7178	0.000
lnURB does not homogeneously cause lnGDP	2.7517	8.4916	0.000
lnGDP does not homogeneously cause lnREN	2.9265	9.3393	0.000
lnREN does not homogeneously cause lnGDP	2.5895	7.7055	0.000
lnGDP does not homogeneously cause lnGOV	4.4455	16.7024	0.000
lnGOV does not homogeneously cause lnGDP	4.7325	18.0940	0.000
lnURB does not homogeneously cause lnREN	13.0420	58.3756	0.000
lnREN does not homogeneously cause lnURB	3.3112	11.2038	0.000
lnURB does not homogeneously cause lnGOV	10.1868	44.5344	0.000
lnGOV does not homogeneously cause lnURB	52.5904	250.0936	0.000
lnREN does not homogeneously cause lnGOV	8.2103	34.9533	0.000
lnGOV does not homogeneously cause lnREN	3.6726	12.9559	0.000

^***^, **, and * represent 1%, 5%, and 10% levels of significance

## Conclusion and policy implications

The Sustainable Development Goal (SDG) 13 highlights the importance of urgent action to combat climate change and its impacts. It is well known that Africa is one of the most vulnerable regions to climate change, and its countries are heavily reliant on agriculture for economic growth, which can be negatively impacted by climate change. To achieve the SDG 13, it is crucial to understand the factors that contribute to climate change, such as CO2 emissions, and how it relates to economic growth, urbanization, renewable energy, and governance in African countries. Using panel data from 1996 to 2019, this study analyzes the dynamic relationships between economic growth, urbanization, and renewable energy, governance, and CO2 emissions across 47 African countries. By examining the relationships between these variables in African countries, this study provides valuable insights into the factors that contribute to greenhouse gas emissions in the region, as well as the potential solutions that can help mitigate climate change. To begin with, we conducted a descriptive analysis of our time-series data, which revealed a positive skewness in our study samples. We also created a spatial map to visualize the average GDP and CO2 emissions per capita for each country in the sample, highlighting significant disparities in the distribution of economic development and carbon emissions across African countries. Subsequently, we carried out a correlation matrix analysis to investigate the relationships between our variables of interest. The results indicate a positive and statistically significant correlation between economic growth, urbanization, governance, and carbon emissions. Moreover, the correlation matrix highlights a negative and statistically significant correlation between renewable energy consumption and carbon emissions.

However, relying solely on the correlation matrix analysis may not be sufficient to draw definitive conclusions about the dynamic effects of our independent variables on the dependent variable. Therefore, we conducted an additional test known as the cross-sectional dependence (CD) test. The purpose of the CD test was to determine the appropriate unit root test to apply. Based on the results obtained, we utilized both first- and second-generation unit root tests to establish the stationarity of our sampled variables. Subsequently, we employed the Pedroni (1999) and Westerlund (2007) cointegration tests to examine the long-run relationships among our variables of interest. Finally, we employed the pooled mean group (PMG) and dynamic fixed effect (DFE) estimators to investigate the short- and long-run impacts among the variables. We also examined the direction of the short-run dynamics between economic growth, urbanization, renewable energy, governance quality, and CO2 emissions using the Dumitrescu-Hurlin causality test. Drawing on the results obtained from the PMG estimator, we arrived at the following conclusions:

1. We found that economic development, as proxied by GDP per capita, has a positive and significant impact on CO2 emissions in Africa. This indicates that as countries in Africa experience economic growth and development, their carbon emissions tend to increase as well. Similar findings are reported by Jayanthi and Merith (2022) and Olusanya and Dasauki (2020)—that an increase in economic growth increases the carbon dioxide emission level of sub-Saharan African countries. Our analysis also reveals that the square of GDP per capita has a negative and statistically significant effect on CO2 emissions in African countries. This finding suggests that the environmental Kuznets curve (EKC) hypothesis holds for the sampled African countries, which means that as countries reach a certain level of economic development, their carbon emissions start to decrease. This finding is consistent with the literature (see Espoir and Sunge 2021), who employed dynamic panel data models to investigate the impact of economic development on CO2 emissions in Africa and found evidence supporting the EKC hypothesis.
2. We document a negative and statistically significant relationship renewable energy consumption and CO2 emissions across African countries. This shows that as the consumption of renewable energy sources increases, the level of CO2 emissions decreases. This is a positive sign for the environment and for efforts to combat climate change, as renewable energy sources have a lower carbon footprint than non-renewable sources such as fossil fuels. It also highlights the potential for African countries to transition to a more sustainable and cleaner energy mix in the future. Our finding aligns with the conclusions drawn by Riti et al (2022) and Ekwueme et al (2021), who found that renewable energy has a significant negative impact on greenhouse gas emissions in Africa and South Africa, respectively.
3. Our study revealed a significant and positive relationship between governance and CO2 emissions in African countries. One possible explanation for this finding is that many African nations may prioritize economic development over environmental concerns, resulting in less stringent environmental policies and regulations. Consequently, the level of carbon emissions may be positively associated with governance indicators. Similar results were reported by Wen et al (2022) and Sarpong and Bein (2020). In contrast, Khan et al. (2022) found a negative relationship between governance and carbon emissions specifically in Morocco. Similarly, Espoir and Sunge (2021) reported a negative impact of governance on carbon emissions in Africa as a whole. These findings suggest that the relationship between governance and carbon emissions may vary depending on the specific context and country being studied.
4. For urbanization, our study’s results suggest that it does not have a significant impact on CO2 emissions in African countries. As stated earlier, one possible explanation for this finding is that the high percentage of urban residents in Africa who experience regular power interruptions may limit the degree to which urban residential energy use contributes to carbon emissions. This phenomenon may be due to factors such as inadequate infrastructure and insufficient investment in the energy sector. As a result, urbanization may not exert a significant impact on CO2 emissions in the context of African countries. The findings are in contrast with the documented results from previous studies conducted by Erdoğan et al (2022) and Musah et al. (2021). These studies found that urbanization worsens environmental degradation in African countries and is a significant contributor to carbon emissions.

The panel cointegration test demonstrated that a long-term relationship exists among the variables. Moreover, the Dumitrescu-Hurlin causality test yielded evidence of bidirectional causality between CO2 emissions and economic growth, urbanization, renewable energy, and governance. The study’s results imply that these factors contribute to carbon emissions in Africa in the short term.

The current research offers several important policy recommendations that merit attention. First, our long-run findings suggest that economic development significantly contributes to carbon emissions in African countries. This shows that economic growth can have negative consequences on the environment, which can ultimately affect the well-being of the population. (1) It is important for policymakers to prioritize sustainable development when promoting economic growth in African countries. Therefore, policies should be designed to encourage the development and implementation of clean and efficient energy resources and waste disposal systems among firms operating in African countries. Governments de-risk investment in renewable energy by providing incentives for companies to invest in renewable energy sources. This can be done by promoting marketing-based instruments like feed-in-tarrifs, tradable pollution permits, and independent power producer (IPPs). Renewable energy sources such as wind, solar, hydropower, and geothermal can provide a clean and sustainable alternative to fossil fuels. In addition to reducing carbon emissions, renewable energy technologies can also help to reduce air pollution, which is a major public health concern in many parts of Africa. At the same time, incorporating the use of renewable energy sources can provide a viable solution that strikes a balance between environmental preservation and sustainable economic growth on the African continent.

Governments could also implement regulations that require firms to reduce their carbon emissions and properly dispose of waste. Additionally, policies could be put in place to encourage the use of energy-efficient technologies and practices. In furtherance of this, governments across the sub-region should promote public–private partnerships that focus on sustainable development. Governments could work with private sector entities to develop and implement sustainable energy and waste management projects. These partnerships could also help to create job opportunities and boost economic growth in the region. (2) The PMG results reveal a significant negative effect of renewable energy consumption on carbon emissions across African countries. However, access to clean energy remains a significant challenge in Africa, and the lack of progress in this area hinders the achievement of SDG 13. Nearly 970 million^3^ Africans lack access to clean cooking, which not only contributes to health problems but also contributes to deforestation and climate change. The high cost of LPG, which is the leading solution in urban areas, further exacerbates the problem. To address this challenge, significant investment is needed in renewable energy sources and infrastructure, particularly in rural areas where access to energy is limited. Governments and international partners such as the Word Bank can play a critical role in providing financing, incentives, and support to promote the adoption of clean energy solutions, including LPG, biogas, and solar.

In addition to improving access to clean energy, efforts must also be made by governments, policymakers, and international development partners such as the World Bank and the African development Bank to promote energy efficiency and conservation across the African continent. This can include measures such as building codes and standards, and public awareness campaigns. (3) Additionally, the long-run findings revealed a significant positive relationship between governance and carbon emissions among African countries. Improving the quality of governance is an essential step in addressing environmental issues and reducing carbon emissions. Governments and policymakers have a critical role to play in this regard, as they can implement policies and regulations that encourage sustainable development and curb carbon dioxide emissions. One way that good governance can contribute to reducing carbon dioxide emissions is by strengthening legal and institutional systems. This can include implementing environmental laws and regulations, establishing effective monitoring and enforcement mechanisms, and ensuring that institutions responsible for environmental compliance are transparent and accountable. Good governance can help to establish effective regulators that can oversee and enforce environmental standards. This can include setting emissions standards for industry, implementing energy efficiency measures, and promoting renewable energy sources. Nevertheless, this requires a commitment from governments and policymakers to prioritize sustainable development and environmental protection, as well as collaboration between stakeholders, including civil society organizations, the private sector, and international partners.

Lastly, the PMG results suggest that urbanization has no significant impact on carbon emissions in African countries. As discussed from the results, economic growth has the tendency to increase carbon dioxide emissions. Conversely, rapid economic growth in African cities also has the potential to stimulate a shift toward clean energy consumption among urban populations, which can lead to a decrease in carbon emissions. However, a shift toward clean energy consumption may not be automatic or immediate and may require supportive policies and incentives from governments and policymakers. While promoting clean energy in urban areas is important, it is also crucial to focus on increasing access to clean energy in rural areas. Therefore, upscaling renewable energy projects in rural areas is an essential step toward promoting sustainable development and reducing carbon emissions in Africa. Policymakers can play a critical role in promoting renewable energy uptake in rural areas by implementing supportive policies and incentives, such as feed-in tariffs, tax incentives, and subsidies. Moreover, efforts to increase access to finance and technology transfer can help to overcome barriers to renewable energy adoption in rural communities. Nevertheless, the finding that urbanization has no significant impact on carbon emissions in African countries highlights the need for more research and analysis in this area. By better understanding the complex relationship between urbanization and carbon emissions, countries can develop more effective policies and strategies to promote sustainable development and reduce emissions in Africa.

## Data Availability

The authors agree to share the data used upon request.

## Appendix

See Table 8

**Table 8:** List of countries and country identifier

Country name	Country identifier	Country name	Country identifier
Benin	1	Namibia	25
Burkina Faso	2	Seychelles	26
Cabo Verde	3	South Africa	27
Côte d'Ivoire	4	Tanzania	28
Gambia	5	Zambia	29
Ghana	6	Zimbabwe	30
Guinea	7	Egypt	31
Guinea-Bissau	8	Burundi	32
Mali	9	Rwanda	33
Niger	10	Libya	34
Nigeria	11	Chad	35
Senegal	12	Tunisia	36
Sierra Leone	13	Algeria	37
Togo	14	Morocco	38
Angola	15	Mauritania	39
Botswana	16	Uganda	40
Comoros	17	Equatorial Guinea	41
DRC	18	Kenya	42
Eswatini	19	Ethiopia	43
Lesotho	20	Gabon	44
Madagascar	21	Cameroon	45
Malawi	22	Congo republic, of	46
Mauritius	23	CAR	47
Mozambique	24		

## Footnotes

1 Brazil, Russia, India, China, and South Africa.

2 We did not report the MG estimates but they can be provided upon request.

3 International Energy Agency (IEA), Africa Energy Outlook 2022 Report.
